# Supplementary material for: Medial geniculate body and primary auditory cortex differentially contribute to striatal sound representations
Source: Nat Commun. 2019 Jan 24;10:418. doi: 10.1038/s41467-019-08350-7 (PMC6346050; doi:10.1038/s41467-019-08350-7)
Supplement: Supplementary file 1 — Supplementary Information [file 41467_2019_8350_MOESM1_ESM.pdf]

Medial geniculate body and primary auditory cortex differentially contribute to striatal sound representations

Chen *et al.*

Supplementary Figure 1

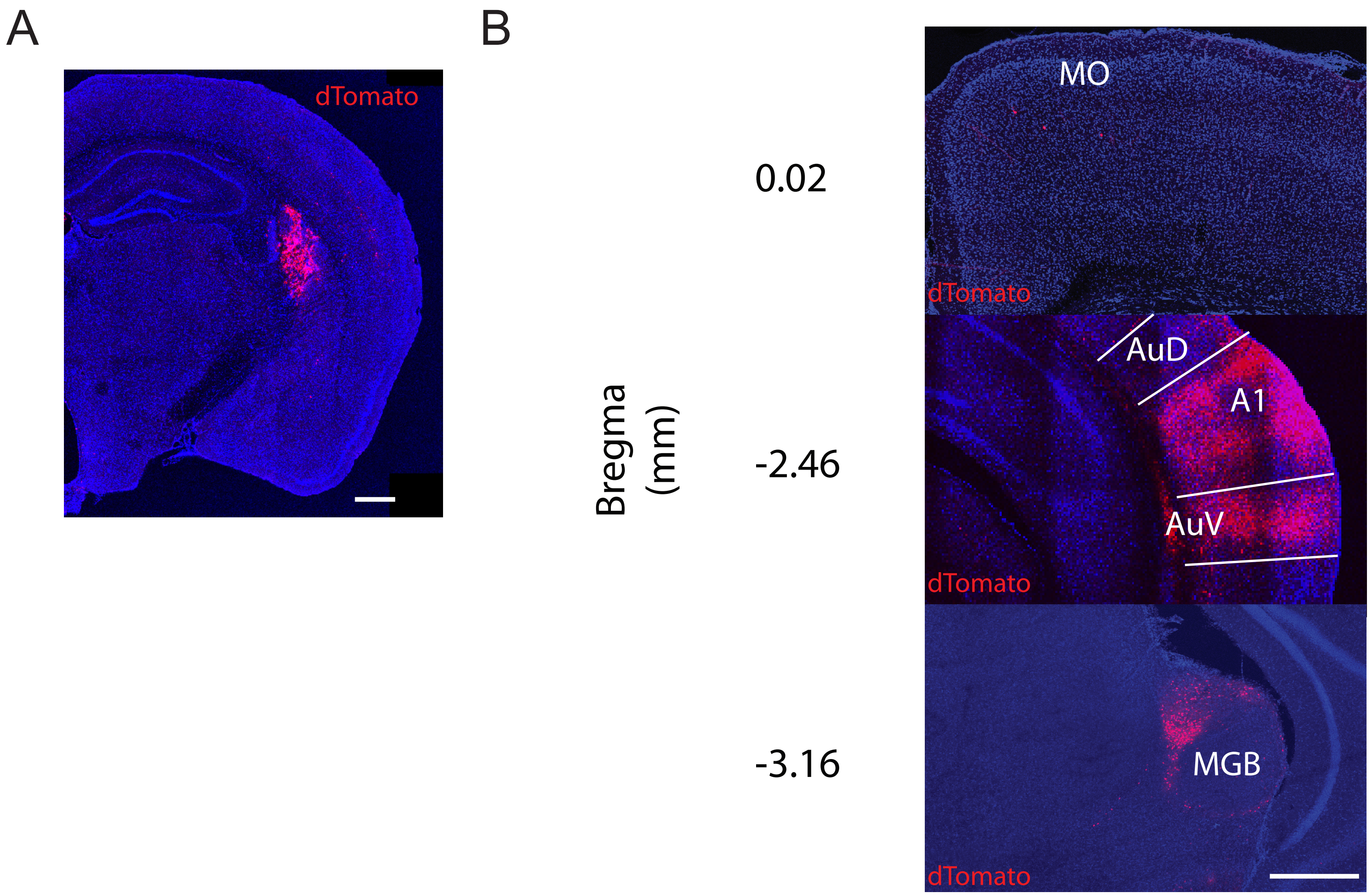

Cortical and subcortical regions projecting to the auditory striatum

A. Upper panel, illustration of virus injection to retrogradely label striatal-projecting neurons. CAV-Cre was injected into the left auditory striatum in Ai14 (FLEX-tdTomato) transgenic mice. Lower panel, an example image showing the injection site. **B.** Sample images of identified regions with tdTomato-positive neurons. MO: motor cortex; A1: primary auditory cortex; AuD: dorsal secondary auditory cortex; AuV: ventral secondary auditory cortex; MGB: medial geniculate body. Scale bars, 500 μm.

Supplementary Figure 2

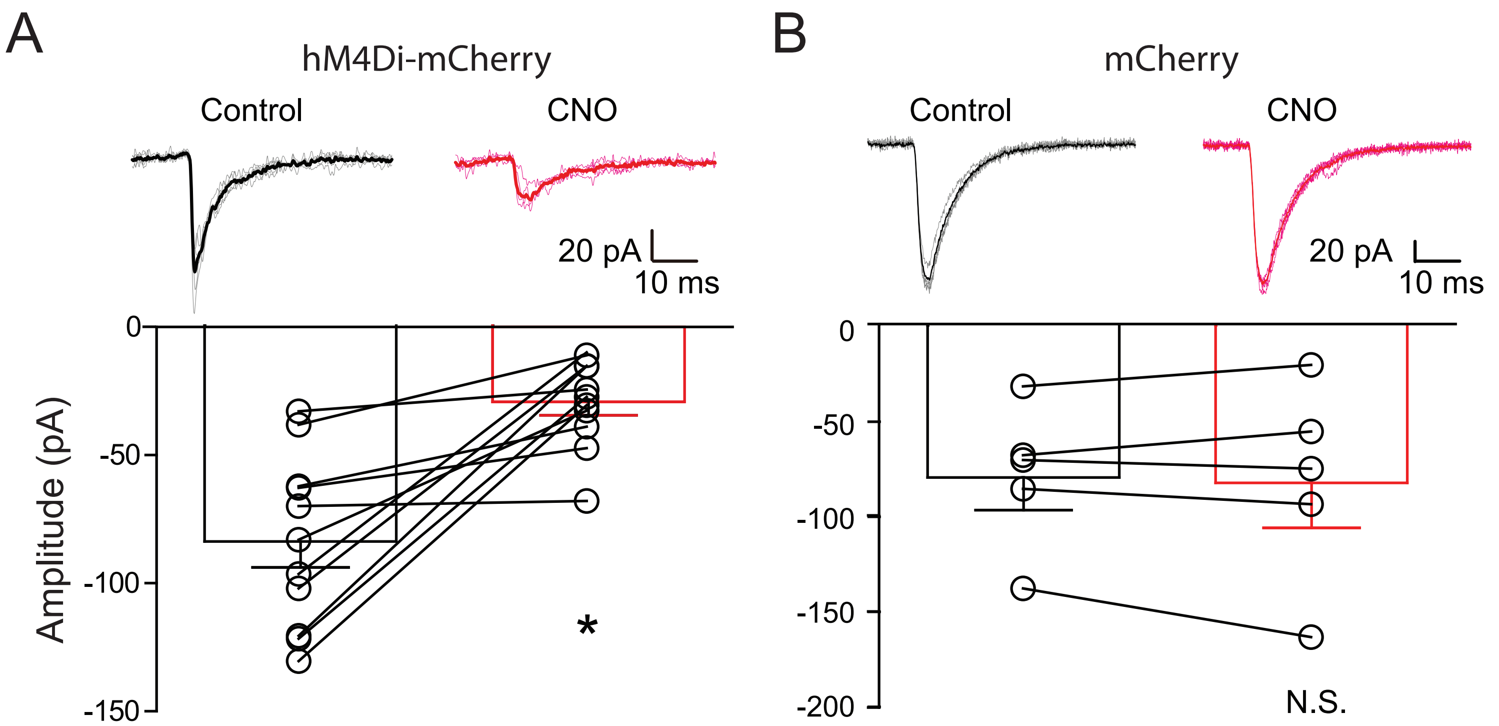

Validation of CNO-hM4Di mediated terminal inhibition.

Validation of CNO-hM4Di mediated MGB terminal inhibition on striatal slices that expressed hM4Di-mCherry (**A**) and mCherry (**B**). Upper panels, example traces of evoked EPSCs with (red) or without (black) CNO (10 μM) application. Lower panels, quantifications of the amplitudes of EPSCs in these conditions. n=11 for both **A&B**, * p=0.0012, paired t-test. Data are presented as mean, error bars are s.e.m.

Supplementary Figure 3

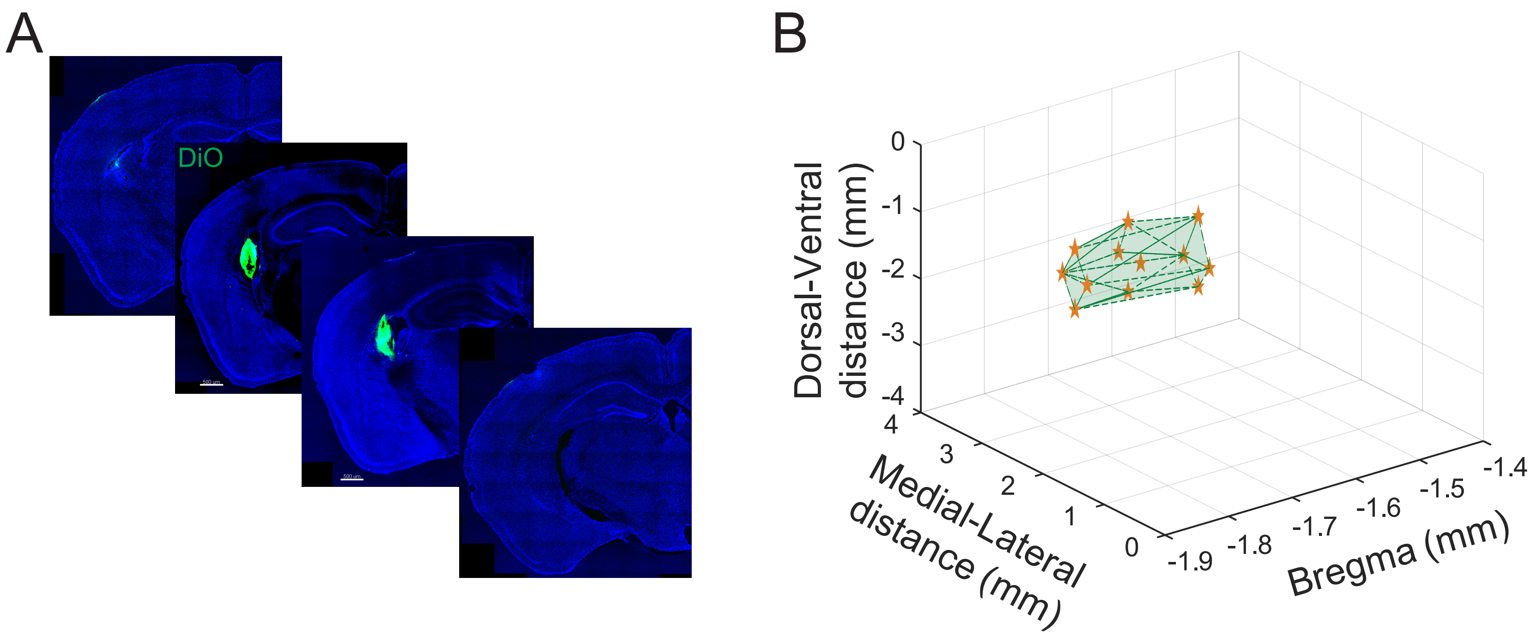

Validation of CNO infusion location and spread.

A. Brain slices with DIO signals for estimation of infusion location spread. To estimate the CNO infusion spread, the same volume of DIO (0.3 μl) was infused through the cannula to label the infusion location and spread. **B.** Quantification of the estimated location and spread from DIO infusion. n= 3 mice.

Supplementary Figure 4

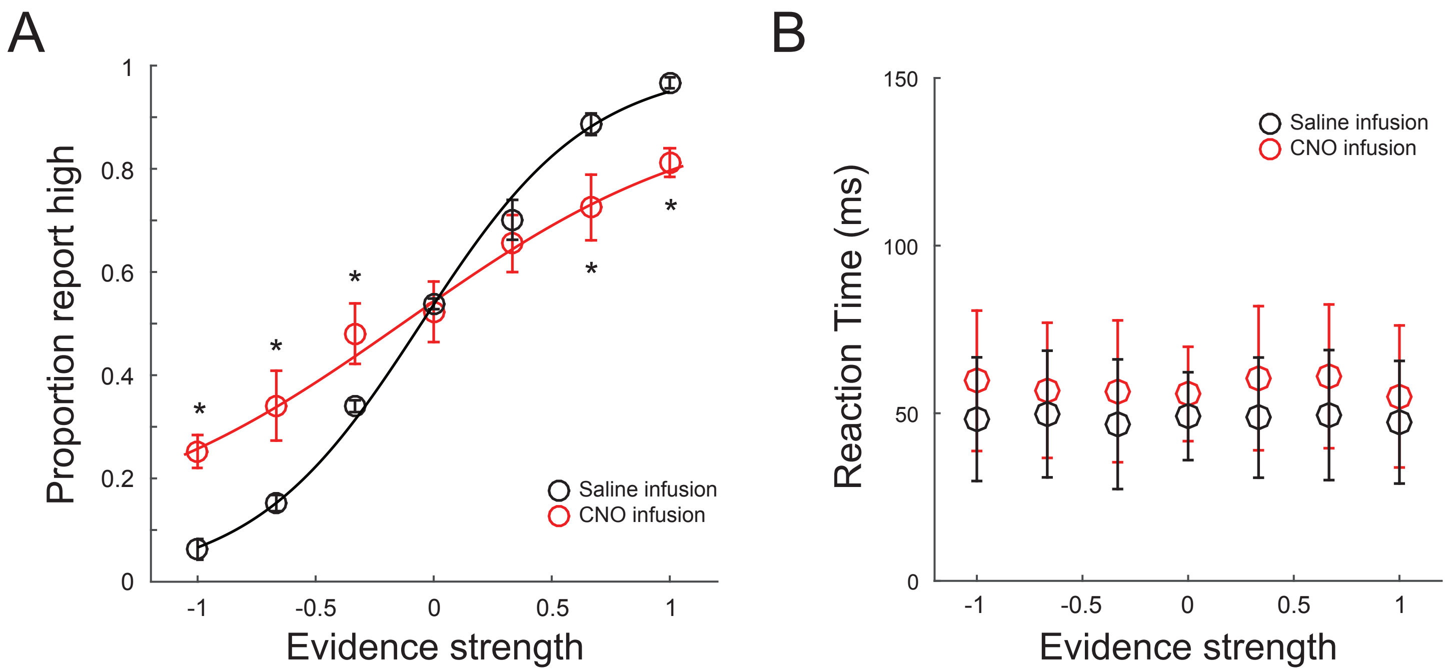

Behavioral effect of inhibiting the primary ACx projection to the auditory striatum.

A. CNO bilateral local infusion effects on task performance in mice expressing hM4Di-mCherry in the primary ACx. The curves were fitted with logistic sigmoid function. Red: CNO sessions; Black: Saline sessions. **B.** Effects of CNO-hM4Di mediated primary auditory cortical inhibition on the reaction time. For **A&B**, n=7 pairs of sessions from 3 mice, Data are presented as mean, and error bars are s.e.m., * $p<0.05$, paired t-test.

Supplementary Figure 5

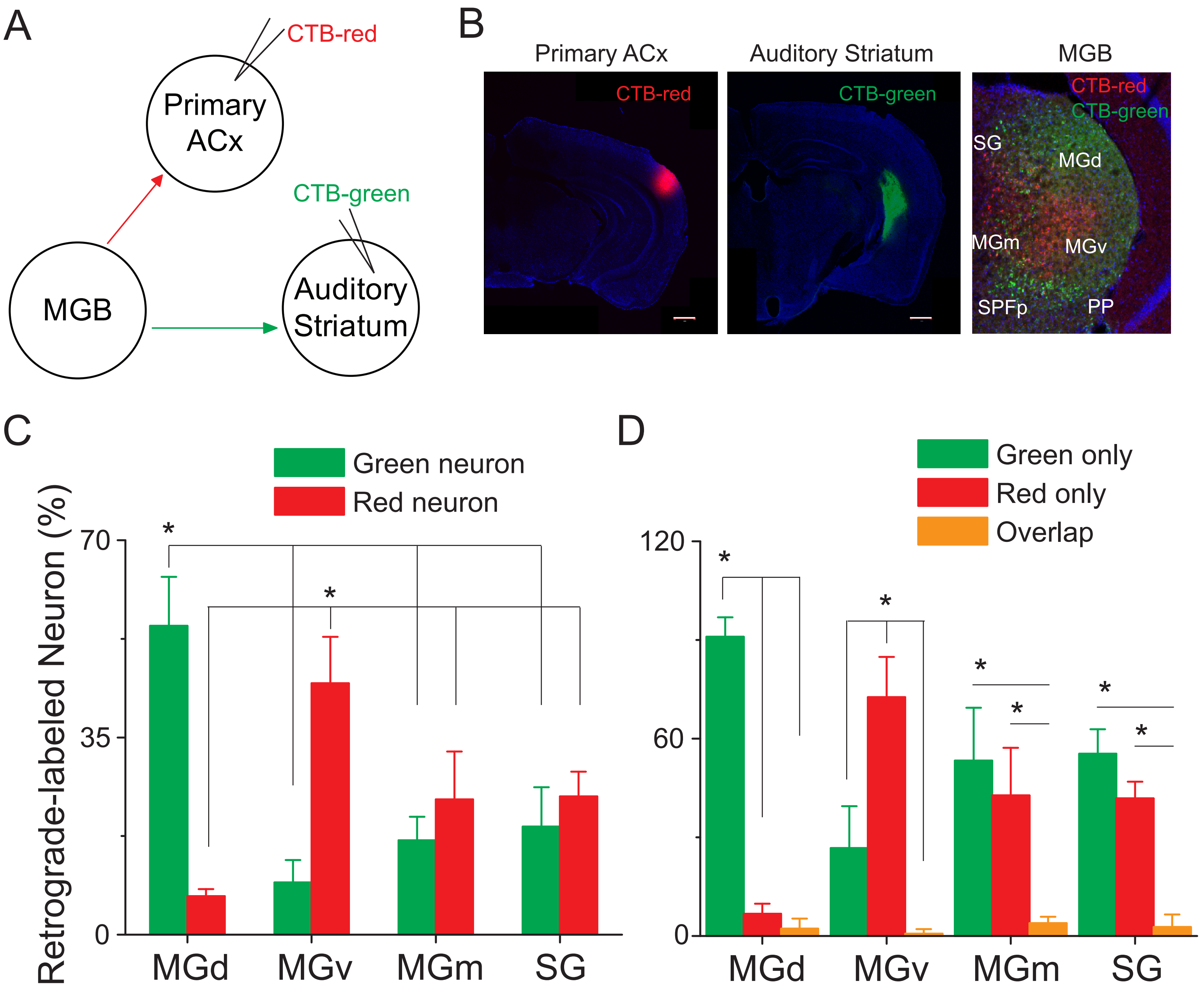

Different MGB neurons project to the primary ACx and the auditory striatum.

A. Injection setup for Cholera toxin B (CTB)-mediated retrograde labeling of MGB neurons. **B.** Fluorophore-conjugated CTB (488 and 594) was injected into the auditory striatum and the primary ACx, respectively (left and middle, scale bar 500 μm) to identify CTB-retrograde-labeled MGB neurons (right, scale bar 200 μm). **C.** Distribution of striatal (green) and primary ACx (red) projecting neurons in MGB: the percentage of labeled neurons in each subdivision divided by the total labeled neurons in MGB. Comparison is within the same color population across the four MGB regions. **D.** The MGB neurons projecting to both cortex and striatum: the percentage of one-color (or double-color) neurons over the total labeled neurons within each MGB subdivision. Green indicates neurons only projecting to striatum, red indicates neurons only projecting to cortex, and orange indicates neurons projecting to both areas. Comparison is within the same sub-region among the three-color populations. For both **C** and **D**, MGd: dorsal MGB; MGv: ventral MGB; MGm: medial MGB; SG: suprageniculate nucleus; PP: peripeduncular nucleus; SPFp: subparafascicular nucleus parvicellular part. n=4 mice for each group; data are presented as mean values, error bars are s.e.m., * $p<0.01$, paired t-test.

Supplementary Figure 6

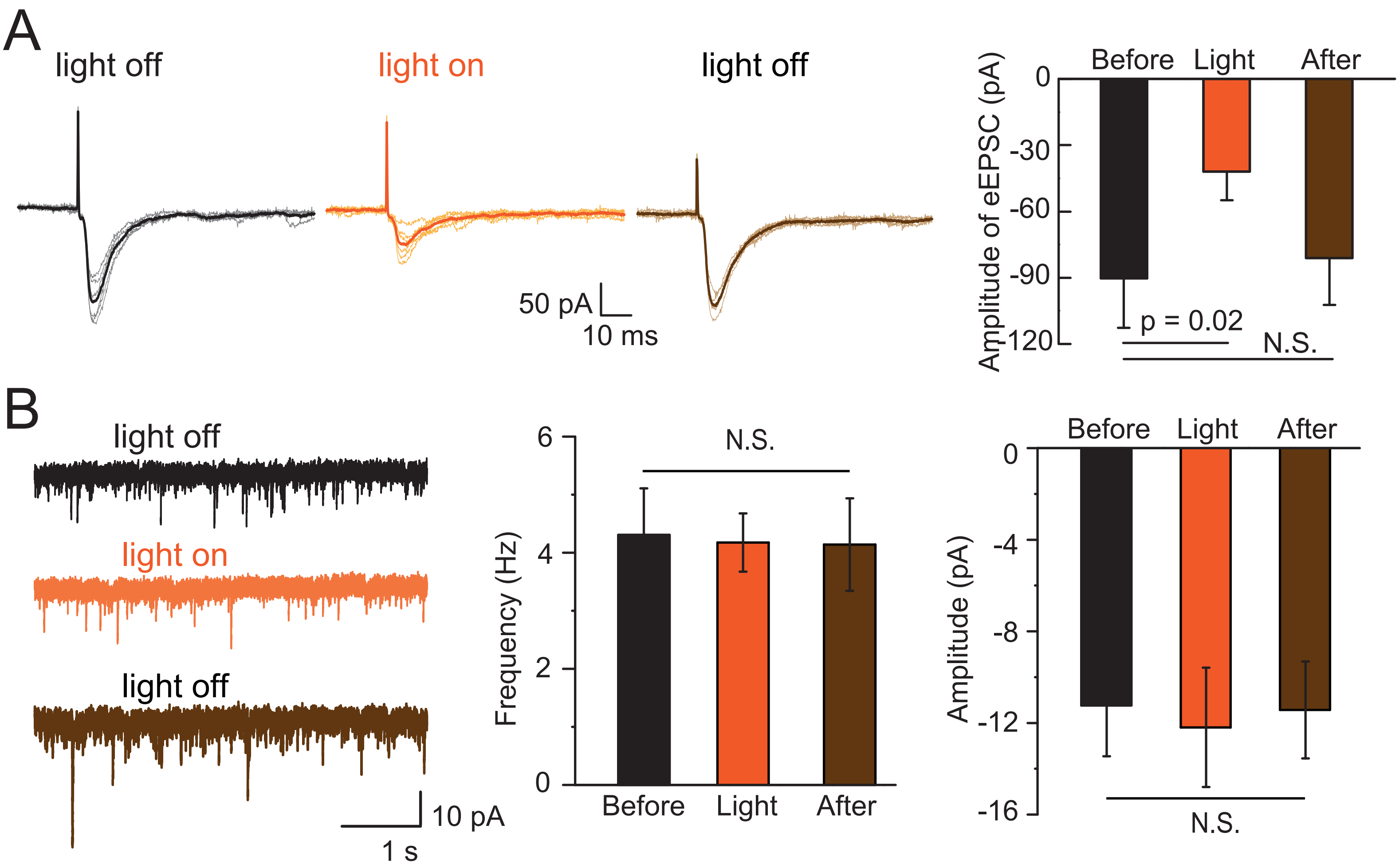

Validation of ArchT-mediated axon terminal silencing.

A. Left panel, example traces of evoked EPSCs recorded from a striatal MSN on brain slice, in the conditions of light on (orange) and light off (black). EPSCs was elicited by local electrical stimulation on axon fibers. Bicuculine (20 μM) was added in the bath solution to block local GABAergic activity. Right panel, quantification of the mean EPSC amplitude at each light condition. n=7, data are presented as mean, error bar is s.e.m. **B**. Left panel, example traces of spontaneous EPSCs recorded from MSN on striatal slice at conditions of light on or light off. Middle and right panels, quantification of frequency and amplitude of the spontaneous EPSCs at each light condition. n=5, data are presented as mean, error bar is s.e.m.

Supplementary Figure 7

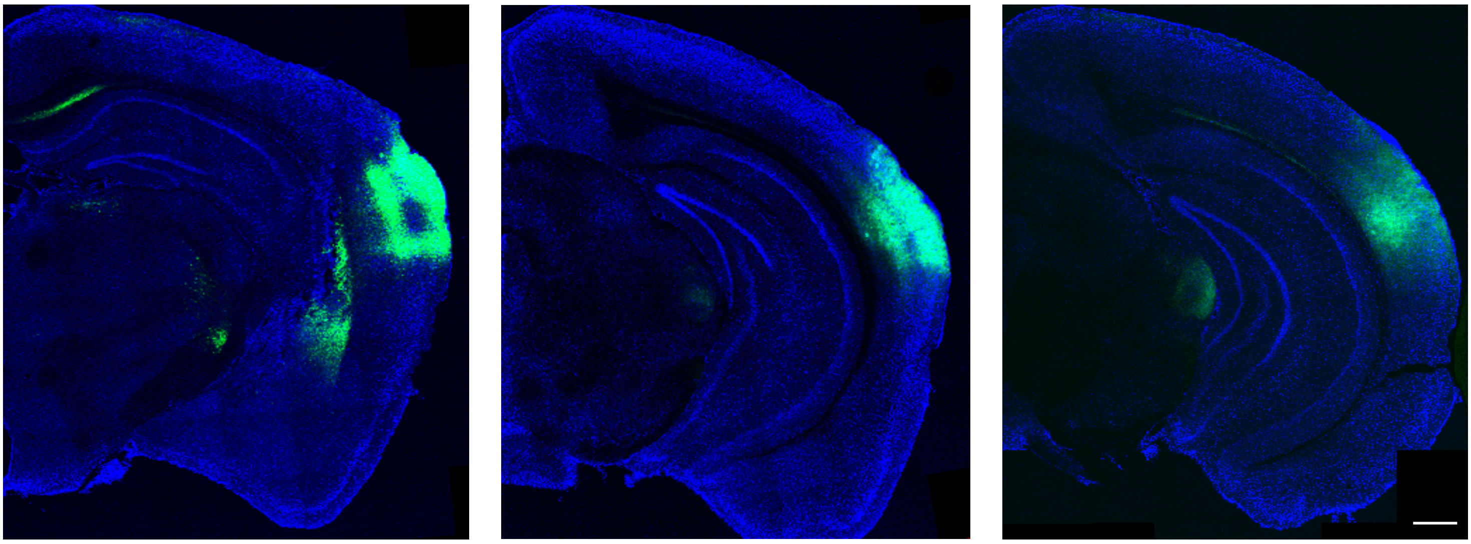

Validation of ArchT expression in the primary ACx.

Three example images of ArchT expression in the primary ACx from one mouse. Scale bar: 500 µm.

Supplementary Figure 8

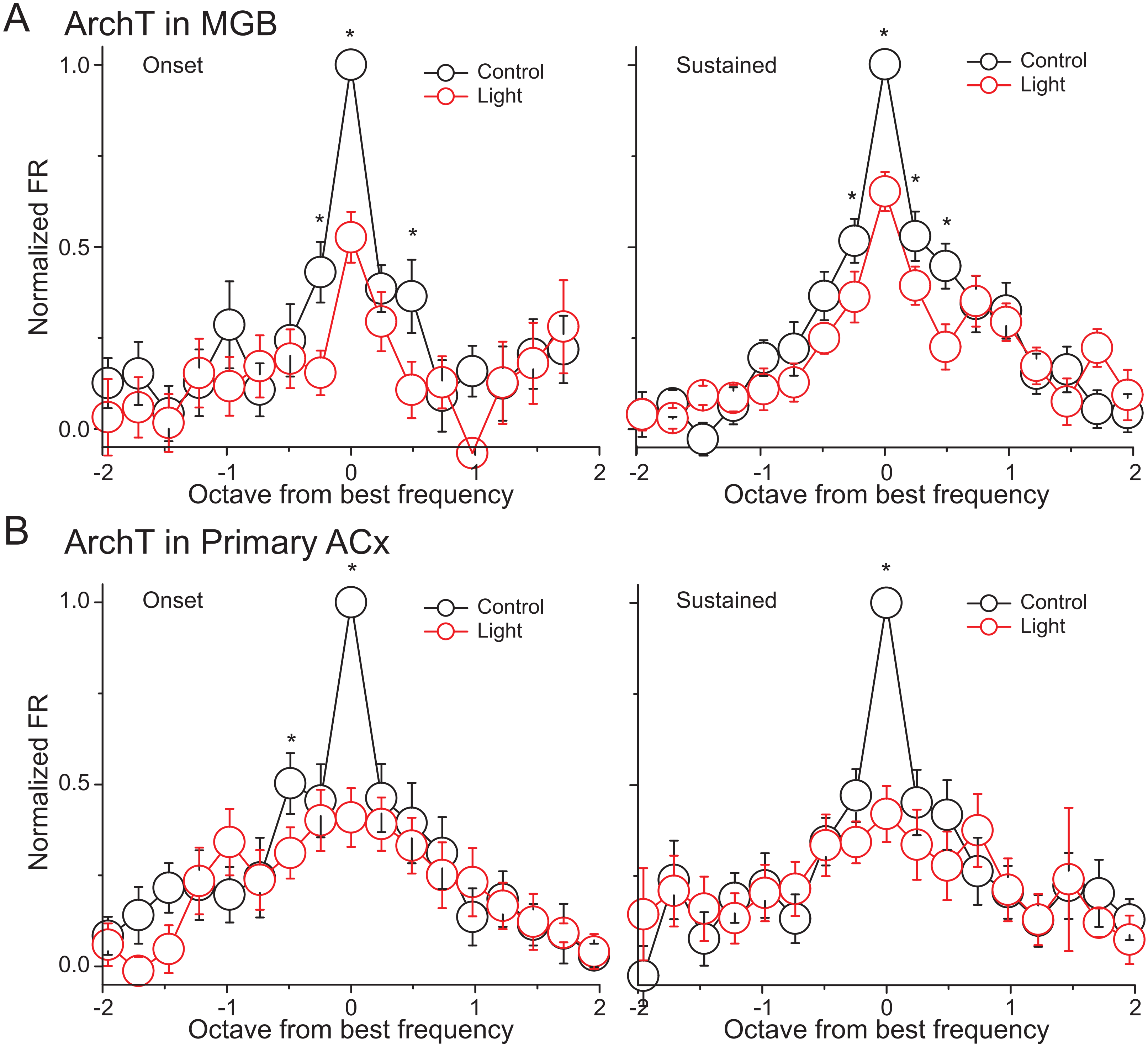

Effects of silencing the MGB or the primary ACx projection on MSNs with onset response and sustained response.

A. Effects of silencing the MGB projection on MSNs with onset response (left panel, n=11) and sustained response (right panel, n=15). **B.** Effects of silencing the primary ACx projection on MSNs with onset response (left panel, n=10) and sustained response (right panel, n=12). Data are presented as mean values, error bars are s.e.m., * $p<0.05$, t-test.

Supplementary Figure 9

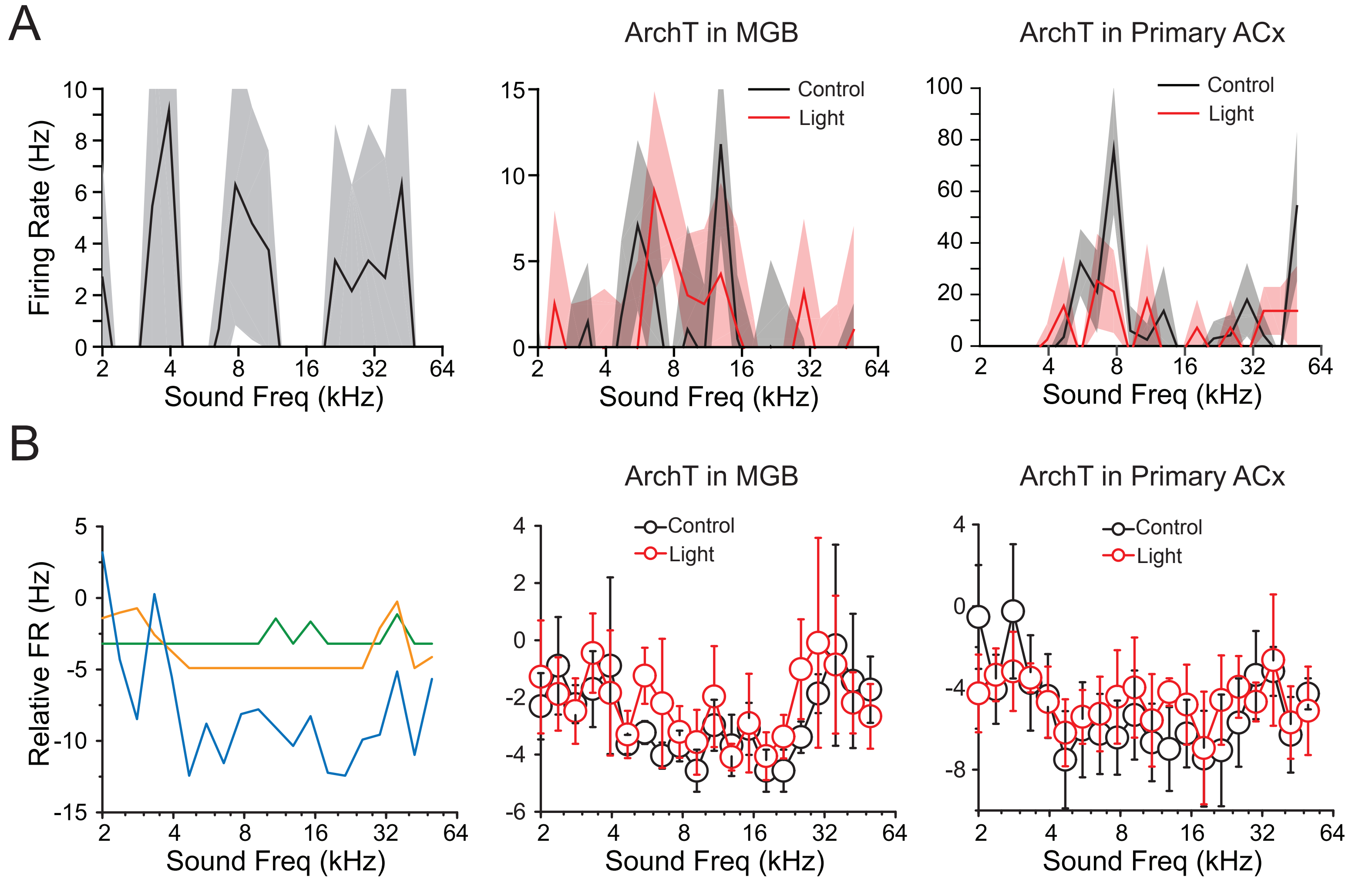

Effects of silencing the MGB or the primary ACx projection on MSNs with offset response and suppression response.

A. The tuning curves of the three offset-responsive MSNs identified from recordings. Left panel, tuning curve of one offset MSN recorded from a control mouse in control condition, middle panel, tuning curve of one offset MSN in control (black) and the MGB projection inhibition (red) condition; right panel, tuning curve of one offset MSN in control (black) and the primary ACx projection inhibition (red) condition. **B.** Left panel, three example tuning curves from the suppression MSNs. Middle panel, averaged tuning curve of the suppression MSNs (n=4) in control (black) and MGB projection inhibition (red) condition; right panel, averaged tuning curve of the suppression MSNs (n=3) in control (black) and the primary ACx projection inhibition (red) condition. Data are presented as mean values, error bars are s.e.m.

Supplementary Figure 10

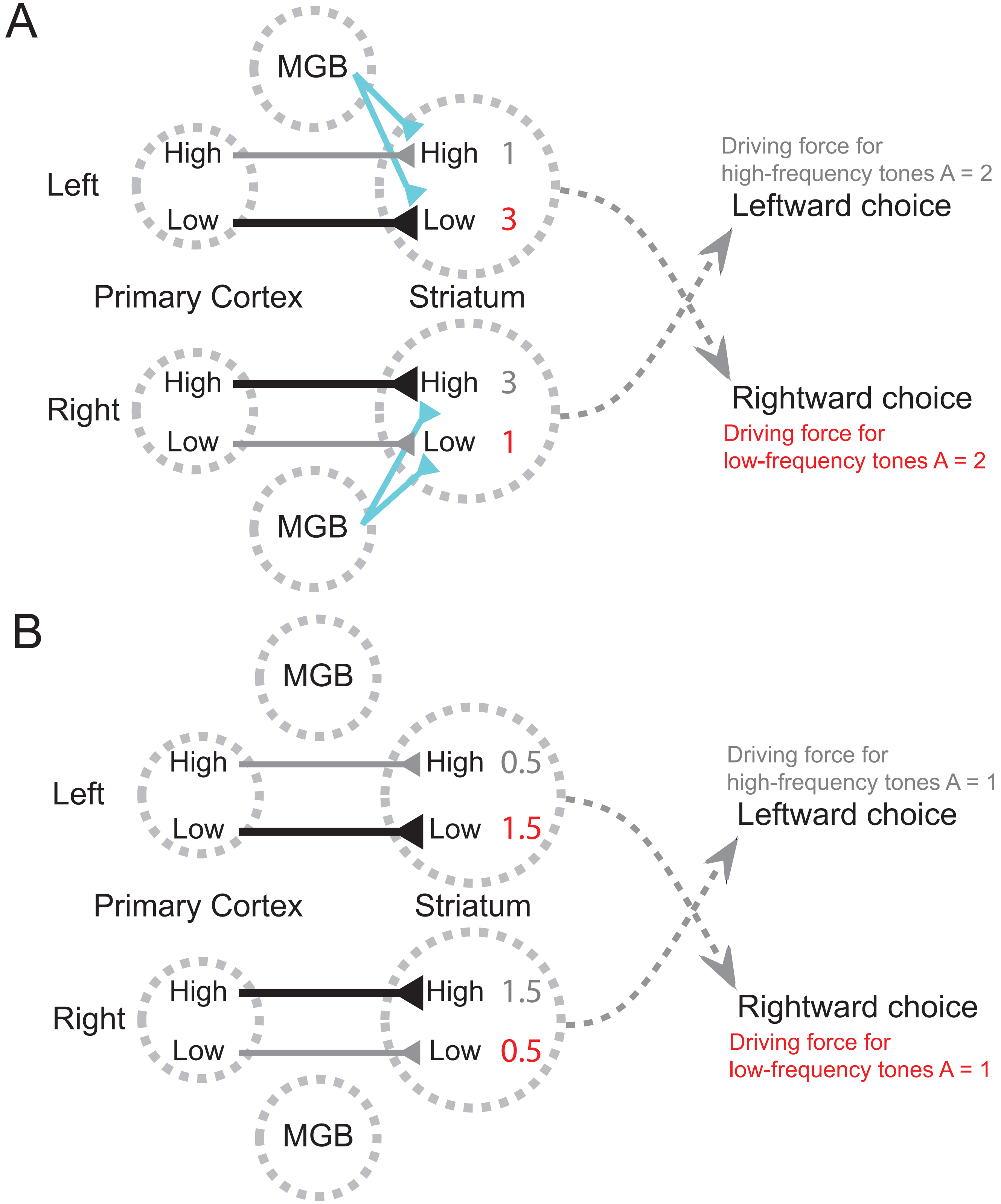

Model showing how the MGB modulation on striatal activity affect behavioral choices in the task.

A. In the control condition from a mouse trained to associate low-frequency tones with rightward choice, corticostriatal synapses tuned to low frequencies in the left hemisphere have stronger synaptic strength than that in the right hemisphere with a driving force A (difference between left and right striatal activity). Thus, low stimuli drive rightward choices. **B.** When the MGB is inhibited, the tone-evoked striatal activity is decreased by a factor of B (for example, 2). In turn the driving force decreases to A/2. The decrease of the driving force may cause the increase of ambiguity during the decision-making, and thus decrease the accuracy in task performance.
